# A Practical Comparison of Ligation-Independent Cloning Techniques

**DOI:** 10.1371/journal.pone.0083888

**Published:** 2013-12-23

**Authors:** Julian Stevenson, James R. Krycer, Lisa Phan, Andrew J. Brown

**Affiliations:** 1 School of Biotechnology and Biomolecular Sciences, The University of New South Wales, Sydney, New South Wales, Australia; 2 Diabetes and Obesity Program, Garvan Institute of Medical Research, Sydney, New South Wales, Australia; Virginia Tech, United States of America

## Abstract

The precise assembly of specific DNA sequences is a critical technique in molecular biology. Traditional cloning techniques use restriction enzymes and ligation of DNA *in vitro*, which can be hampered by a lack of appropriate restriction-sites and inefficient enzymatic steps. A number of ligation-independent cloning techniques have been developed, including polymerase incomplete primer extension (PIPE) cloning, sequence and ligation-independent cloning (SLIC), and overlap extension cloning (OEC). These strategies rely on the generation of complementary overhangs by DNA polymerase, without requiring specific restriction sites or ligation, and achieve high efficiencies in a fraction of the time at low cost. Here, we outline and optimise these techniques and identify important factors to guide cloning project design, including avoiding PCR artefacts such as primer-dimers and vector plasmid background. Experiments made use of a common reporter vector and a set of modular primers to clone DNA fragments of increasing size. Overall, PIPE achieved cloning efficiencies of ∼95% with few manipulations, whereas SLIC provided a much higher number of transformants, but required additional steps. Our data suggest that for small inserts (<1.5 kb), OEC is a good option, requiring only two new primers, but performs poorly for larger inserts. These ligation-independent cloning approaches constitute an essential part of the researcher's molecular-tool kit.

## Introduction

**Figure 1:**
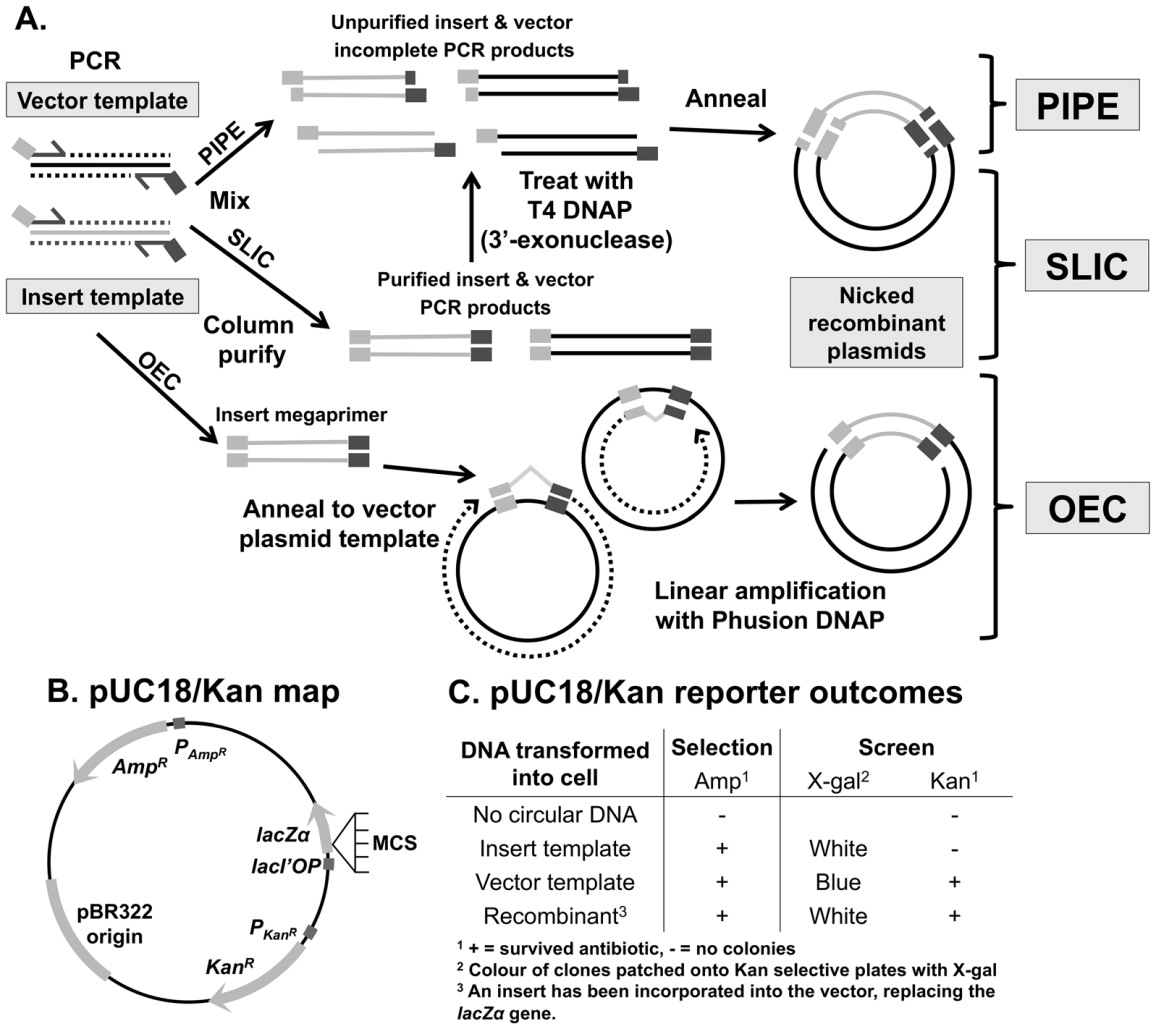
Principles of polymerase incomplete primer extension (PIPE) cloning, sequence and ligation-independent cloning (SLIC) and overlap extension cloning (OEC). (A) In PIPE, incomplete extension during PCR generates 3′-recessed ends. In SLIC, purified PCR products are treated with T4 DNA polymerase (DNAP) so that the exonuclease activity will increase the proportion of recessed ends. In both these techniques, by amplifying vector and insert with primers containing complementary 5′-tails and mixing the products, the overhangs can anneal and are joined *in vivo* after transformation into *E. coli.* In OEC, the insert PCR product acts a megaprimer to generate a nicked plasmid by overlap extension *in vitro* in a second round of amplification. The nicks are also repaired *in vivo*. For all techniques, a DpnI digestion step is included to remove plasmid template (not shown). (B) Design of the reporter vector, encoding resistance for ampicillin and kanamycin, and the alpha-fragment of beta-galactosidase. (C) Colonies from ampicillin plates were patched onto kanamycin/X-gal plates to distinguish recombinants from unwanted insert vector or empty pUC18/Kan background colonies.

The precise assembly of specific DNA sequences is a critical technique in molecular biology. Traditional cloning makes use of restriction enzymes and ligation of DNA *in vitro*. Restriction endonuclease digestion and ligation increase the complexity of cloning projects, for example by requiring selection of appropriate restriction-sites and inefficient ligation steps. Consequently, several ligation-independent cloning (LIC) methods have since been developed that are simpler, faster, and highly efficient. These strategies rely on the generation of DNA fragments with single-stranded complementary ends to allow directional cloning of any insert, independent of restriction enzymes and *in vitro* ligation. The most effective and convenient methods include polymerase incomplete primer extension (PIPE) cloning [1], sequence and ligation-independent cloning (SLIC) [2], and overlap extension cloning (OEC) [3], [4] (Figure 1). In this study, we will compare these cloning strategies.

All three techniques amplify the gene of interest by polymerase chain reaction (PCR). The 3′ ends of the PCR primers are template-specific, whilst the 5′ ends incorporate tails specific for the cloning junction.

PIPE relies on the observation that a significant portion of PCR products are incomplete, having 3′-recessed ends [1], particularly in the absence of a final extension step. SLIC uses a brief treatment of purified PCR products with the 3′→5′ exonuclease activity of T4 DNA polymerase to generate a higher proportion of recessed ends [2], [5]. Cloning is possible due to the complementary 5′ ends of the insert and vector fragments. These are analogous to the much shorter ‘sticky ends’ generated by restriction enzymes. After mixing, these single-stranded overhangs anneal and can be efficiently ligated *in vivo* after transformation by the bacterial DNA recombination and repair machinery.

In contrast, OEC has two rounds of amplification. Firstly, the insert is PCR-amplified using primers with 5′ ends complementary to the target site in a circular destination vector. The 3′ ends of this product (‘megaprimer’) can consequently anneal and amplify the destination vector by overlap extension. This yields a nicked plasmid that is repaired after transformation. A variant of the OEC strategy is utilised by the commonly used QuikChange site-directed mutagenesis kit (Agilent Technologies).

In an age of high-throughput molecular biology, there is a need to move away from traditional cloning methods. We believe that LIC methods offer a more robust, efficient means for DNA cloning. Furthermore, variations between strategies suggest that certain techniques may be better suited for particular situations. Consequently, here we characterise and compare the efficiency, convenience, and utility of three major LIC techniques.

## Materials and Methods

### Primer combinations

Primer sequences are listed in Table S1 in File S1. Gene accession numbers and plasmid vector backbones are listed in Table S2 in File S1. The pUC18/Kan reporter vector (Figure 1B) was prepared using PIPE cloning. The vector backbone of pUC18 was obtained by PCR with primers pUC18–F and pUC18–R, and the kanamycin resistance cassette from pEGFP-C1 (Clontech) with primers KanR-in-pUC-F and KanR-in-pUC-R. The complete pUC18/Kan vector DNA sequence is included as a text file in Supporting Information (File S2).

A 24 bp FLAG epitope insertion (84 bp fragment) was achieved through PIPE or SLIC with primers FLAG-pUC18-F and FLAG-pUC18-R to amplify pUC18/Kan. The 85 bp FLAG megaprimer for OEC was prepared through 40 uL thermal cycling of the primers alone, with 4 µM FLAG-pUC18-overlap-F and FLAG-pUC18-overlap-R with 1× HF Buffer, 0.8 U of Phusion Hot Start II High-Fidelity DNA Polymerase (New England Biolabs) and cycling conditions: (98°C 30 s, 63°C 30 s, 72°C 1 min) ×5.

The pUC18/Kan vector backbone was amplified with primers pUC18-L30-F and pUC18-L30-R, which are the reverse complement of the common 5′-tails of the insert primers. A 350 bp fragment (Gluc) was amplified from FLAG-hLXRβ-hGluc(1) [6] with primers Gluc-pUC18-F and BGH-pUC18-R. LXRβ (1.4 kb) was similar amplified with primers LXRb-pUC18-F and LXRb-pUC18-R. A 2.2 kb sequence (AR) from pTK-AR-V5 [6] was obtained with primers AR-pUC18-F and AR-pUC18-R. SRC-1 (4.3 kb) was amplified with primers SRC1-pUC18-F and SRC1-pUC18-R from pCR3.1-SRC1 [7]. 1.4 kb Insig-1 and 4.3 kb SCAP fragments were obtained with primers T7-pUC18-F and BGH-pUC18-R from pCMV-Insig-1-Myc or pCMV-SCAP respectively [8].

### PCR

PCR was performed in 50 µL reactions using 0.5 ng of template, 0.5 µM forward and reverse primers, 5% DMSO, HF Buffer and 1 U of the non-strand displacing enzyme Phusion Hot Start II High Fidelity DNA polymerase, and cycling conditions: 98°C 3 min, (98°C 30 s, 63°C 30 s, 72°C 3 min for products >1.5 kb or 1 min for <1.5 kb) ×30.

Reaction products were column purified using a QIAquick PCR purification or gel extraction kit (Qiagen) and quantified by spectroscopy.

### PIPE cloning

0.025 pmol of vector and 0.0625 pmol insert purified products (2.5∶1 insert∶vector ratio) were digested for 3 hr at 37°C with 10 U of DpnI in 10 µL CutSmart Buffer (NEBuffer 4.1/0.1 mg/mL BSA), diluted 1∶1 with 1× HF buffer (to control for buffer composition between purified and unpurified products, as 1× HF buffer halved transformation efficiency), and 2 µL used to transform 18 µL of XL10 Gold ultracompetent cells (Agilent Technologies) according to manufacturer's instructions.

### SLIC

DpnI digested purified PIPE products were incubated at 25°C for 5 min with 0.75 U of T4 DNA polymerase, immediately placed on ice for 10 min, diluted 1∶1 with ice-cold 1× HF Buffer, and 2 µL used for transformation as described above.

### OEC

20 µL OEC reactions were performed using 10–250 fmol of insert product −84 bp: 250 fmol (12.5 nM); 350 bp: 100 fmol (5 nM); 1.4 kb: 25 fmol (1.25 nM); 4.3 kb: 10 fmol (0.5 nM) - as megaprimer and 25 ng of pUC18/Kan vector template with 5% DMSO, 0.4 U of Phusion Hot Start II High Fidelity DNA polymerase, and cycling conditions: 72°C 5 min (to blunt the megaprimer), 98°C 3 min, (98°C 30 s 63°C 30 s 72°C 3 min) ×30. 5 µL fractions were diluted 1∶1 with CutSmart Buffer (to control for buffer and assist DpnI activity), digested for 3 hr at 37°C with 20 U of DpnI, and 2 µL used for transformation as described above.

### Colony counting and screening

Serial dilutions of transformation mixture were spread onto ampicillin selective plates to allow counting of the number of colony forming units (CFU), adjusted to account for total volume, and rounded to 3 significant figures. 40 colonies were picked and streaked onto kanamycin selective plates in the presence of X-gal/IPTG to identify recombinants. For one replicate experiment of each set, colonies were also tested by colony PCR across the cloning junctions or sequenced to validate the blue/white screening.

**Step by step protocols for each technique are outlined in the supporting information** (**File S3**).

## Results

### Design of the reporter system

**Figure 2:**
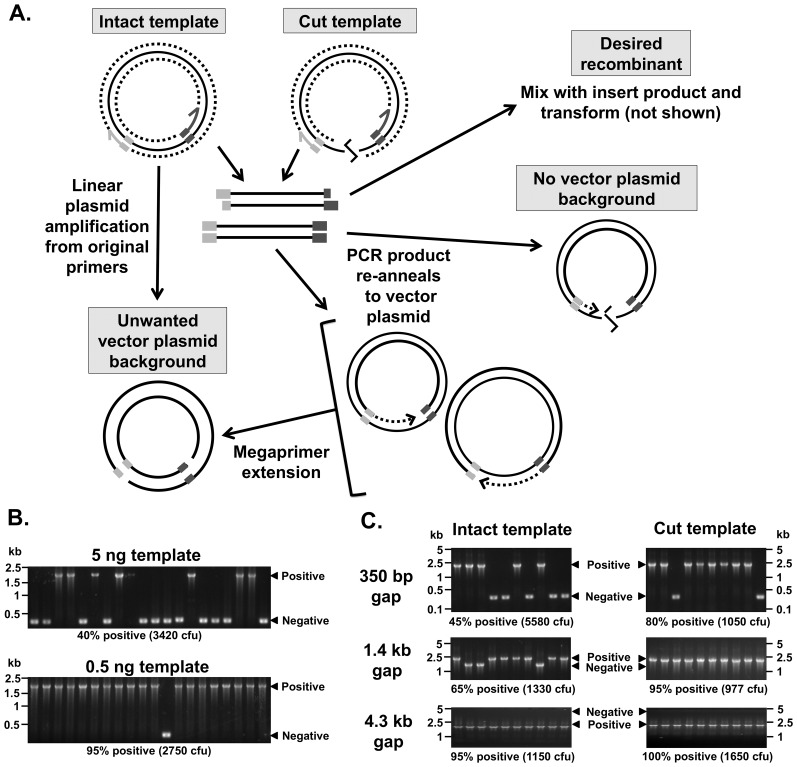
Generation of nicked vector plasmid can reduce PIPE cloning efficiency. Unwanted copies of the original vector plasmid template can be generated by overlap extension in the process of obtaining vector-backbone PCR product (A). This can be prevented by reducing the template concentration (B), cutting the plasmid template first or cloning into a vector with an existing large insert (C). See main text for details.

To efficiently compare the different cloning techniques, we prepared a reporter vector plasmid, pUC18/Kan (Figure 1B). It contains resistance cassettes for ampicillin and kanamycin, as well as a polylinker encoding the *lacZα* fragment. Each pair of insert-specific primers had the same 30 bp 5′-tail – this modular design allowed replacement of the multiple-cloning site of pUC18/Kan with a collection of inserts. This allows simple identification of the type of plasmid in each colony – insert plasmid, vector plasmid or desired recombinant plasmid – as the insert plasmids lack kanamycin resistance, and the vector plasmid template contains undisrupted *lacZα*. Hence, bacteria were first plated onto ampicillin plates and then patched onto kanamycin selective plates in the presence of IPTG for blue/white colony screening. Thus, positive colonies are white and resistant to both ampicillin and kanamycin (Figure 2C), whilst blue colonies possess pUC18/Kan and Kan-sensitive colonies possess insert-containing plasmids.

### Generation of nicked vector plasmid reduces PIPE cloning efficiency

We began our optimisation with PIPE. After first transforming the DpnI-digested vector PCR product alone we observed a significant number of blue (empty pUC18/Kan) colonies, but no colonies without the PCR (data not shown). Given that DpnI only digests methylated DNA, this indicates that vector plasmid is being generated during the PCR. This can occur because extension of the vector primers all the way around the plasmid template can regenerate the empty vector plasmid (Figure 2A). Note that this is linear amplification, generating less product in contrast to the desired PCR product between the primers, which is amplified exponentially by PCR. However, the vector PCR products can also re-anneal to the vector and extend analogously to the megaprimers in OEC, although this appeared to be less important, as addition of three 5′ non-template thymidines to the vector primers did not affect cloning efficiency (data not shown). Nevertheless, these situations can generate nicked copies of the vector *in vitro* that will escape DpnI digestion (Figure 2A). This leads to unwanted vector background colonies.

To confirm this mechanism, we hypothesised that nicked vector background should be reduced by 1) increasing the gap between the 5′ ends of the vector primers, or 2) cutting the template in between the vector primers (Figure 2). To test this, we replaced 350 bp, 1.4 kb, or 4.3 kb inserts in pUC18/Kan (i.e. different gap sizes) with the same 2.2 kb insert. The insert primer 5′ vector overhangs were complementary to the vector backbone, independent of the pre-existing insert sequences. This allows us to determine PIPE cloning efficiency – the proportion of successful recombinants - based on insert size differences using colony PCR. This was performed with or without linearising the vector using PstI. We found that both increasing the gap and linearisation reduced background (Fig 2C). However, because these strategies restrict the utility of PIPE-cloning, we sought a more flexible solution. We reasoned that reducing the template should dilute out the background – since the complete vector is linearly amplified, it is more dependent on template concentration and thus a lower template concentration would favour PCR amplification of the desired product. Accordingly, PIPE cloning efficiency increased from approximately 40% to greater than 95% when using ten-fold less template (Figure 2B), with similar results for insertion of the 350 bp, 1.4 kb and 4.3 kb inserts into pUC18/Kan (Table S3 in File S1). We adopted this latter strategy in subsequent experiments.

### Optimisation of SLIC, PIPE and OEC

PIPE relies on incomplete extension in PCR. Although the proportion of incomplete products is sufficient at 25 cycles [1], we reasoned that additional cycles might deplete PCR components or generate DNA products that inhibit extension, leading to a greater proportion of recessed ends and thus more clones. However, PIPE cloning efficiency did not increase using equal amounts of DNA product taken from 25 to 40 PCR cycles (Table S4 & S5 in File S1). We maintained subsequent reactions at 35 cycles to ensure that good product yields are achieved, particularly for difficult templates or inefficient primers. PIPE cloning tolerated a wide range of insert∶vector molar ratios for a range of insert sizes (Table S6 in File S1), with an optimum of 2.5∶1, similar to previously reported values for SLIC [2], [5].

We made use of a one-tube version of SLIC [5], [9]. For a convenient volume of T4 polymerase (0.75 U/0.25 µL), the highest efficiency was observed after 5–10 min treatment at 25°C, although 5 min was most robust (Table S7 in File S1), followed by immediate incubation on ice to halt the reaction.

OEC uses linear amplification, generating less product than the exponential amplification in PCR. We consequently used 25 ng of template for OEC, 50-fold higher than for the PCR-based PIPE and SLIC. This required doubling the concentration of DpnI to account for the increased template. The effectiveness of OEC was dependent upon megaprimer concentration, with the optimum being inversely proportional to megaprimer size: high concentrations of small insert and vice versa (Table S8 in File S1). Thus concentrations of ∼25–50 fmol (1.25–2.5 nM) were optimal for products >1.5 kb, ∼50–100 fmol (2.5–5 nM) <1.5 kb, and ∼100–300 fmol (5–15 nM) <350 bp. This is likely due to the increasing propensity of larger products to anneal to themselves rather than to the plasmid template. We observed more kanamycin-sensitive colonies when the megaprimer was originally prepared from insert PCRs with higher template (5 ng). This is likely to be insert-vector background from the insert PCR, and was removed by reducing the template amount to 0.5 ng (Table S9 in File S1). Increasing the number of cycles of overlap extension gave progressively more colonies, without clearly increasing cloning efficiency (Table S10 in File S1). Hence 30 thermal cycles were used for comparisons to the other techniques.

### OEC does not tolerate the presence of primer-dimers

**Figure 3:**
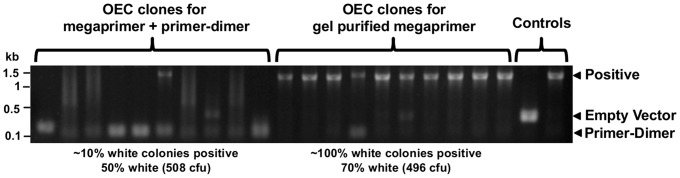
OEC does not tolerate primer-dimers. Megaprimer and primer-dimer contaminant or 1.4 kb LXR megaprimer alone were gel purified and used for OEC. Ten white colonies for each were screened by PCR across the cloning junctions.

To test the ability of the techniques to tolerate the presence of primer-dimer, we attempted to clone a 1.4 kb gene product where the PCR product also included a marked amount of ∼150 bp primer-dimer or mispriming product. Since these products contain 5′ ends complementary to the vector, they may be incorporated into recombinant clones, also yielding white colonies in our screening assay. Primer-dimer had little effect on PIPE or SLIC, as screened white colonies contained the correct insert, confirmed by colony PCR and sequencing (data not shown). In contrast, for OEC, most white colonies contained unwanted primer-dimer instead (Figure 3). Prior gel extraction of the insert megaprimer and using this in the overlap extension step ensured that nearly all white colonies contained the desired insert instead (Figure 3). An alternative method to remove primer-dimer before OEC was to pretreat the purified insert mixture with T4 DNA polymerase in the absence of dNTPs (Figure S1 in File S1). This allowed digestion of the primer-dimer, leaving the larger desired product to be end-filled - during the 5 min, 72°C step at the start of the overlap extension reaction - and cloned.

### Direct comparison

To directly compare the effectiveness of the different cloning strategies, the reporter vector and different inserts were first amplified and purified, then fed into PIPE, SLIC and OEC with equivalent amounts of DNA using the optimised protocols (as described in the Materials and Methods). Reaction products were diluted with either HF buffer or NEBuffer to control for transformation buffer composition, as the Phusion 1× HF PCR buffer halved transformation efficiency (data not shown). The amount of DNA (25 fmol vector and 62.5 fmol insert) used for transformation was equivalent to that of unpurified PIPE cloning from a single pair of PCRs. 24 bp, 350 bp, 1.4 kb and 4.3 kb fragments were chosen to gauge the effect of increasing size on cloning efficiency for each strategy.

The 24 bp insertion is a special case due to its small size, in that PIPE and SLIC used a single primer pair of reverse design for PCR – with 3′ ends complementary to the vector and 5′-tails containing the entire insert – thus requiring only one PCR, such that the product anneals to itself to form a circular product. For very small inserts, using two primer sets to amplify vector and insert would be wasteful and very inefficient for PIPE and SLIC. The standard insert primer design was used for OEC, with annealing and 3′ end-filling of partially overlapping primers alone to first prepare the insert megaprimer.

PIPE consistently performed well, yielding hundreds of colonies with close to 100% cloning efficiency (Tables 1, S6 and S10). Addition of T4 DNA polymerase exonuclease treatment for SLIC increased the number of transformants by ∼4–100 fold for all inserts, retaining the high efficiency.

**Table 1:** Direct comparison of PIPE, SLIC and OEC for various insert sizes.

Insert Size^a^	Technique	Cloning Efficiency	Colonies	Fold Increase^b^
85 bp	PIPE	100%	1560	1
	SLIC	100%	15800	10
	OEC	95%	15100	10
350 bp	PIPE	98%	176	1
	SLIC	98%	18500	105
	OEC	90%	9200	52
1.4 kb	PIPE	95%	705	1
	SLIC	100%	2760	4
	OEC	73%	1450	2
4.3 kb	PIPE	100%	309	1
	SLIC	100%	6420	21
	OEC	45%	251	1

See main text for details.

^a^ Single representative experiments are shown for FLAG (85 bp), Gluc (350 bp), Insig-1 (1.4 kb), and SCAP (4.3 kb).

^b^ The increase in colonies relative to PIPE is shown.

OEC yielded very high numbers of transformants for the 24 bp (84 bp megaprimer) insertion, with close to 100% efficiency. Moderate to high efficiencies were also observed for the 350 bp insertion. Colony number fell proportionally as insert size increased, which was associated with a corresponding decline in cloning efficiency (Table 1). Cloning efficiencies and colony numbers were far more variable for larger fragments, reflecting lower robustness of OEC compared to the other LIC techniques (Table S8–S11 in File S1).

## Discussion

Ligation-independent cloning techniques can be used to introduce DNA fragments into cloning vector plasmids quickly, cheaply, and with high efficiency. Anything that can be amplified by PCR can be introduced into any position of any vector of choice in a single cloning step without unwanted additional nucleotides, so called ‘scarless cloning’. We observed that PIPE worked very well with limited manipulations, as long as the template concentration was kept to a minimum to avoid overlap extension vector background. SLIC consistently achieved the highest efficiencies and number of transformants, but required additional resources. OEC worked well for smaller fragments, but was less effective for larger fragments. It was also very vulnerable to the presence of primer-dimers.

The methods compared here work either through the generation of complementary single-stranded overhangs for *in vivo* homologous recombination (PIPE, SLIC) or by generating a nicked plasmid *in vitro* by overlap extension (OEC). This can require the design of new primers with longer complementary tail sequences than used for restriction cloning, but the time savings and increased robustness outweigh these nominal costs, even for routine applications. Modular primers can also be designed to allow parallel cloning of a gene into different vectors with identical linkers [10], as well as inserting different genes with the same 5′-tails into the same vector, as in this study.

**Figure 4:**
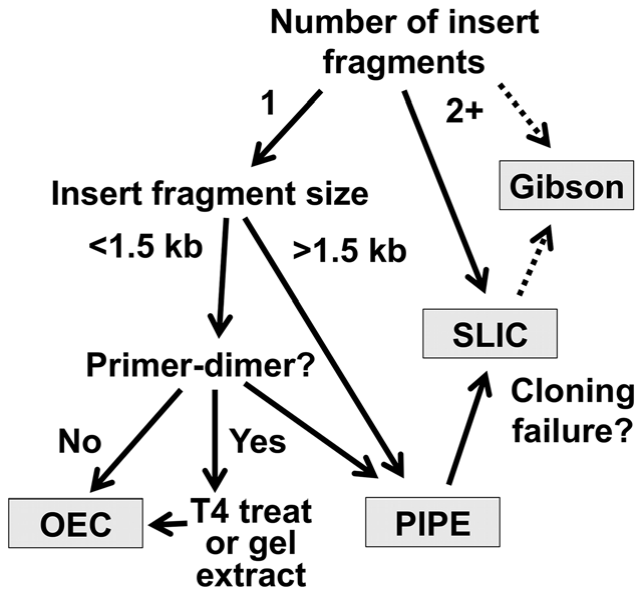
Technique selection flowchart for a new cloning project. The number of fragments, their size, primer availability and the presence of primer-dimers will determine the optimal cloning strategy. See main text for details.

The identical insert primer design also allowed the use of the same PCR product with the three different techniques. This can enable rescue of a failed cloning attempt using a different strategy with a higher efficiency but requiring additional steps and resources (Figure 4 and Table 2). The first method of choice is dependent upon the nature of the insert, the availability of existing primers and the level of efficiency required. For genes of up to 1.5 kb, OEC is a good choice, as useful efficiencies (50%+) can be achieved with one primer pair. This will often be suitable for insertion of affinity tags or fluorescent proteins, such as the green fluorescent protein. However, cloning efficiency and robustness for OEC decrease with increasing insert size, introducing the risk of failure for larger genes. Overlap extension also requires relatively long (∼30 bp) target-vector-tail sequences in the primers to allow stable annealing, whereas PIPE and SLIC can work well with overhangs of ∼15 bp, allowing synthesis of shorter primers [1], [2], [5]. Purification of the insert PCR is recommended for OEC, but for smaller inserts, a wide range of megaprimer concentrations are tolerated, safely allowing use of a small fraction of unpurified PCR product as megaprimer (Table S12 in File S1 and data not shown). PIPE cloning is generally simpler and robustly performs well, at the cost of requiring a second primer pair.

**Table 2:** Summary of effectiveness and resource use.

LIC Techniques	PIPE	SLIC	OEC
Cloning Efficiency <1.5 kb	Very High	Very High	High
Colony Number <1.5 kb	High	Very High	High
Cloning Efficiency >1.5 kb	Very High	Very High	Low
Colony Number >1.5 kb	High	Very High	Low
Primer Pairs	2	2^a^	1
Purification	Optional	Yes	Yes^b^
Thermal Cycling Rounds	1	1	2
T4 Treatment Step	No	Yes	No

See main text for details.

^a^ Only 1 primer pair is required for SLIC if cleaved plasmid vector is used.

^b^ Optional for small megaprimers (∼500 bp).

For cloning projects where vector primers are already available or higher efficiencies are desired to clone inserts greater than 1.5 kb, PIPE is usually the best choice, achieving high efficiencies for a wide range of gene sizes [1] (Table 1 and Table S11 in File S1). Unpurified PIPE PCR products can be used for transformation directly after DpnI treatment, making it extremely fast, especially useful for high-throughput cloning projects [1]. This simple approach is the mainstay LIC technique in our laboratory and our default choice for any fragment size (See protocols in File S3). However, purification to remove inhibitory buffer components and concentrate the DNA provides better results (Table S12 in File S1 and data not shown). If PIPE cloning fails or a greater number of transformants are required, such as for library construction, then SLIC is superior, typically increasing the number of transformants by more than 5-fold in one step after purification, DpnI and T4 exonuclease treatment. SLIC is also effective after combining purified fragments without quantification (Table S12 in File S1). SLIC can also be performed using restriction enzyme-cleaved plasmid, minimising primer design at the cost of additional manipulations over PIPE [2], [5], [9].

SLIC has the advantage that it can be used to assemble multiple insert fragments in a single cloning step, particularly with addition of recombinant RecA to enhance annealing *in vitro*, albeit with reduced efficiency compared to single-fragment cloning [2]. This can also be achieved with high efficiency using the Gibson method [11], which uses similar primer design to PIPE and SLIC, but involves use of a 5′ exonuclease, DNA ligase and DNA polymerase *in vitro* to join previously generated PCR fragments. This has proved to be particularly useful for synthetic biology projects requiring assembly of very large DNA fragments. However, for single fragment insertion, we do not believe that the increased resources required are justified when PIPE and SLIC robustly achieve high efficiencies. However, Gibson assembly [11] can prove a highly effective alternative when even SLIC fails (Figure 4).

A number of commercial cloning kits make use of site-specific recombination, requiring special vectors and costly proprietary enzymes, such as the Gateway® system (Life Technologies). Heterostagger or mixed PCR cloning only requires primers and polymerase to precisely engineer recessed ends similar to PIPE and SLIC [2], [12]. Although very effective, it is also costly as it requires double the number of PCRs and primers used in SLIC, and long denaturing and annealing steps, without performing better [2].

We found that PIPE, SLIC and OEC were also very efficient for site-directed mutagenesis. PIPE or SLIC are optimal for large deletions [1], [10], and extremely effective for substitutions or small insertions like epitope tags (Table 1). However, to create point mutations we routinely use an alternative two-stage cycling strategy similar to OEC, adapted from QuikChange [13], [14]. It uses one mutagenic primer and a shorter pre-existing one, often a sequencing primer, to create a megaprimer by PCR, which then generates the mutant plasmid by overlap extension. This normally provides adequate efficiencies at minimal expense.

The LIC techniques tested in this study can be highly efficient and technically simple, but can be compromised by problems such as nicked vector plasmid background, cloning of primer-dimers and amplification of non-specific PCR products. However, these can be overcome through careful design, checking PCR products, and taking additional measures accordingly. Primer-dimers and non-specific products are only likely to be a problem if visible on the agarose gel or with a Bioanalyzer. However, nicked copies of the vector are often below the limit of detection, yet can still generate significant unwanted background (data not shown). Vector background colonies are generated when the vector plasmid templates are amplified rather than just the insert, or desired vector-backbone product (Figure 2). This potential problem is most relevant to PIPE, due to a relatively low number of transformants. The simplest effective method to reduce this background for PIPE is to use less template. If that fails or if greater robustness is desired, a restriction enzyme can be used to first cut the template outside of the primer binding sites. Increasing the gap between the primers was also beneficial for our pUC18/Kan reporter, likely due to the difficulty of amplifying larger products in their entirety. We have previously observed higher efficiencies when replacing genes rather than PIPE cloning into an empty vector [6]. Using a vector containing an existing insert of distinguishable size can be particularly beneficial if it includes the lethal *ccdB* gene to select against vector background [1].

Non-specific PCR products and primer-dimers can be an important problem. Primer-dimers had little effect on PIPE or SLIC, likely due to their small size, such that they would lack the 5′-tails required for single-strand annealing and recombination. However, even low concentrations of primer-dimer are of concern for OEC, since cloning is extremely efficient for very small inserts, which clone preferentially. Primer-dimers could be removed by gel extraction or through pre-incubation with T4 polymerase to digest primer-dimer. Larger non-specific products will also compete with the desired insert in PIPE and SLIC, reducing cloning efficiency, which may require gel extraction. In our experience, these non-specific products can also be reduced through design of longer 3′ ends, increasing annealing temperatures, and reducing primer concentration. To avoid the requirement for gel extraction or PCR optimisation, alternative primer design can be used for Quick and Clean Cloning [9], a more specific variant of SLIC. This involves designing longer primers for one of the cloning junctions where the vector overhang is complementary to the region within the target insert PCR product, rather than insert primer. The resulting fragments can still be joined because short (∼20 bp) ends of non-complementary sequence adjacent to the desired sequence are removed and successful recombination can occur *in vivo*, as long as the internal complementary sequence is single-stranded [2], [9].

Ligation-independent cloning approaches constitute an essential part of the biomedical researcher's molecular-tool kit. With the extremely high fidelity of modern polymerases and availability of modular vector-specific primers, we find that ligation-independent methods are preferable even for simple subcloning projects. Due to their robustness, speed and low cost, they may largely supplant restriction enzyme and ligation-dependent cloning in many laboratories.

## Supporting Information

File S1 **Supporting Figure and Tables.**
(PDF)

File S2 **pUC18/Kan Reporter Vector Sequence.**
(TXT)

File S3 **Supporting Protocols.**
(PDF)
