# Supplementary material for: A Practical Comparison of Ligation-Independent Cloning Techniques
Source: PLoS One. 2013 Dec 23;8(12):e83888. doi: 10.1371/journal.pone.0083888 (PMC3871625; doi:10.1371/journal.pone.0083888)
Supplement: File S1 — Supporting Figure and Tables. (PDF) [file pone.0083888.s001.pdf]

A Practical Comparison of Ligation-Independent Cloning Techniques

Julian Stevenson[1, 3,*], James R. Krycer[1,2,3,*], Lisa Phan[1], and Andrew J. Brown[1,*]
[1] School of Biotechnology and Biomolecular Sciences, The University of New South Wales, Sydney, NSW, 2052, Australia
[2] Diabetes and Obesity Program, Garvan Institute of Medical Research, Sydney, NSW, 2010, Australia
[3] These authors contributed equally to this work

* To whom correspondence should be addressed. Tel: +61-2-9385-2005; Fax: +61-2-9385-1483; Email: aj.brown@unsw.edu.au
Correspondence may also be addressed to Julian Stevenson; Email: j.stevenson@unsw.edu.au or James R. Krycer; Email: j.krycer@garvan.org.au

SUPPORTING FIGURE AND TABLES

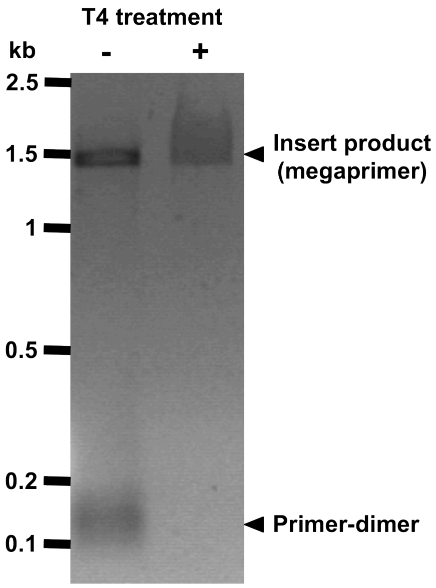

Figure S1. Primer-dimer can be removed with T4 DNA polymerase exonuclease treatment. 0.25 pmol of 1.4 kb LXR purified PCR product was treated for 30 min at 25 °C with 3 U of T4 DNA polymerase.

Table S1. Primer sequences. Vector tail sequences are indicated by boldface. Overlapping reverse-complementary sequences are underlined.

Primer Name	Primer Sequence (5' → 3')
KanR-in-pUC-F	**CGTGCCAGCTGCATTAATGAATCGGCCAAC**GCGGAACCCCTATTTGTTTA
KanR-in-pUC-R	**CGAGCGCAGCGAGTCAGTGAGCGAGGAAGC**TCATTTCGAACCCCAGAGTC
pUC18–F	GCTTCCTCGCTCACTGACTC
pUC18–R	GTTGGCCGATTCATTAATGC
pUC18-L30-F	CGAGCTCGAATTCGTAATCATGGTCATAGC
pUC18-L30-R	CGCATATGGTGCACTCTCAGTACAATCTGC
FLAG-pUC18-F	GACTACAAGGACGACGACGACAAA**CGAGCTCGAATTCGTAATCATGGTCATAGC**
FLAG-pUC18-R	TTTGTCGTCGTCGTCCTTGTAGTC**CGCATATGGTGCACTCTCAGTACAATCTGC**
FLAG-pUC18-overlap-F	**GCAGATTGTACTGAGAGTGCACCATATGCG**GACTACAAGGACGACGACGACAAA
FLAG-pUC18-overlap-R	**GCTATGACCATGATTACGAATTCGAGCTCG**TTTGTCGTCGTCGTCCTTGTAGTC
LXRb-pUC18-F	**GCAGATTGTACTGAGAGTGCACCATATGCG**TCCTCTCCTACCACGAGTTC
LXRb-pUC18-R	**GCTATGACCATGATTACGAATTCGAGCTCG**CTCGTGGACGTCCCAGATCT
Gluc-pUC18-F	**GCAGATTGTACTGAGAGTGCACCATATGCG**CAACGAAGACTTCAACATCGTG
BGH-pUC18-R	**GCTATGACCATGATTACGAATTCGAGCTCG**TAGAAGGCACAGTCGAGG
T7-pUC18-F	**GCAGATTGTACTGAGAGTGCACCATATGCG**CGCAAATGGGCGGTAGGCGTG
SRC1-pUC18-F	**GCAGATTGTACTGAGAGTGCACCATATGCG**AGTGGCCTCGGGGACAGTT
SRC1-pUC18-R	**GCTATGACCATGATTACGAATTCGAGCTCG**TTCAGTCAGTAGCTGCTGAAGGA
AR-pUC18-F	**GCAGATTGTACTGAGAGTGCACCATATGCG**ATGGAAGTGCAGTTAGGGCTGGGAA
AR-pUC18-R	**GCTATGACCATGATTACGAATTCGAGCTCG**CTGGGTGTGGAAATAGATGG

Table S2. Plasmid names and gene accession numbers. Gene sequences can be found on the NCBI GenBank or RefSeq databases. Vector backbone information is available on the Addgene Vector Database (http://www.addgene.org/vector-database/).

Plasmid	Gene	NCBI Gene Accession Number	Vector Backbone	Reference
pTK-AR-V5	AR	NM_000044.3	pcDNA3.1D/V5-His-TOPO	[6]
FLAG-hLXRβ-hGluc(1)	hGluc(1)	HQ388295.1	pCMX	[6]
	hLXRβ	NM_007121.5		
pCMV-Insig-1-Myc	Insig-1	NM_005542	pcDNA3	[8]
pCMV-SCAP	SCAP	NM_012235.2	pcDNA3	[8]
pCR3.1-SRC-1	SRC-1	NM_003743.4	pCR3.1	[7]
pUC18/Kan	See supporting .txt file for complete vector sequence and main text for plasmid map.			Current work

Table S3. Effect of template concentration on PIPE cloning efficiency. Vector products amplified from 5 ng or 0.5 ng of template per 50 μl reaction were combined with purified insert at a 5:1 I:V ratio. 40 colonies were patched onto kanamycin/X-gal/IPTG plates to screen for potential recombinants (white colonies), rather than empty vector (blue colonies) or insert vector (kanamycin sensitive, Kan (-), colonies). Cloning efficiency is the percentage of successful recombinants.

Template	Insert	Colonies	Cloning Efficiency	White	Blue	Kan (-)
5 ng	350 bp	2420	28%	11	29	0
	1.4 kb	1620	30%	12	28	0
	4.3 kb	1070	8%	3	37	0
0.5 ng	350 bp	967	88%	35	3	2
	1.4 kb	814	93%	37	2	1
	4.3 kb	140	88%	35	4	1

Table S4. Cycle number for insert product does not affect PIPE cloning efficiency. Insert products purified from 25-40 cycle reactions were combined at a 5:1 I:V ratio with 40 cycle vector product.

Insert	Exp. #	Cycles	Colonies	Cloning Efficiency	White	Blue	Kan (-)
350 bp	1	25	1080	63%	25	14	1
		30	830	78%	31	9	0
		35	935	65%	26	14	0
		40	880	68%	27	13	0
	2	25	2220	83%	33	5	2
		30	2610	73%	29	10	1
		35	1860	60%	24	13	2
		40	2600	78%	31	8	1
	3	25	1550	50%	20	24	0
		30	1450	58%	23	19	0
		35	2430	73%	29	11	0
		40	2200	63%	25	15	0
1.4 kb	1	25	1200	80%	32	8	0
		30	1510	93%	37	3	0
		35	1510	78%	31	9	0
		40	1230	88%	35	5	0
	2	25	685	48%	19	21	0
		30	830	48%	19	20	1
		35	1130	68%	27	12	1
		40	1520	78%	31	9	0
	3	25	646	78%	31	6	3
		30	1400	75%	30	9	1
		35	1140	85%	34	4	2
		40	1020	80%	32	7	1
4.3 kb	1	25	715	85%	34	6	0
		30	600	80%	32	8	0
		35	605	68%	27	13	0
		40	600	75%	30	10	0
	2	25	330	40%	16	24	0
		30	357	45%	18	22	0
		35	961	75%	30	10	0
		40	1960	95%	38	2	0
	3	25	655	85%	34	2	4
		30	740	73%	29	10	1
		35	860	78%	31	8	1
		40	940	80%	32	7	1

Table S5. Cycle number for vector product does not affect PIPE cloning efficiency. Vector products purified from 25-40 cycle reactions were combined at a 5:1 I:V ratio with 40 cycle insert products.

Insert	Cycles	Colonies	Cloning Efficiency	White	Blue	Kan (-)
1.4 kb	25	2450	93%	37	2	1
	30	2560	88%	35	5	0
	35	3560	88%	35	5	0
	40	3360	88%	35	5	0
4.3 kb	25	638	95%	38	2	0
	30	660	88%	35	5	0
	35	605	93%	37	3	0
	40	412	75%	30	10	0

Table S6. Effect of insert:vector ratio on PIPE cloning efficiency. Purified products were combined at the indicated molar ratios.

Insert	Exp. #	I:V	Colonies	Cloning Efficiency	White	Blue	Kan (-)
350 bp	1	1:1	585	40%	16	24	0
		2.5:1	1180	75%	30	9	1
		5:1	1250	80%	32	8	0
		10:1	2240	90%	36	4	0
1.4 kb	1	1:1	2700	68%	27	13	0
		2.5:1	2490	90%	36	4	0
		5:1	2420	90%	36	4	0
		7.5:1	3380	90%	36	4	0
		10:1	3290	93%	37	3	0
	2	1:1	1600	90%	36	4	0
		2.5:1	1980	93%	37	3	0
		3:1	3300	83%	33	3	0
		5:1	3170	88%	35	5	0
		10:1	2400	85%	34	6	0
	3	1:1	775	90%	36	4	0
		2.5:1	1190	95%	38	2	0
		5:1	785	100%	40	0	0
		7.5:1	975	88%	35	4	1
		10:1	720	80%	32	8	0
4.3 kb	1	1:1	516	78%	31	9	0
		2.5:1	715	90%	36	4	0
		5:1	620	75%	30	10	0
		10:1	500	85%	34	6	0

Table S7. Effect of T4 exonuclease treatment time on colony number. 0.025 pmol of vector and 0.0625 pmol insert purified products were digested with DpnI then treated for 5 or 10 min with 0.75 U of T4 DNA polymerase, as described in the Materials and Methods. Untreated (0 min) conditions are identical to PIPE cloning.

Insert	Exp. #	T4 treatment (min)	Colonies	Cloning Efficiency	White	Blue	Kan (-)
85 bp	1	0	2650	98%	39	0	1
		5	77700	100%	40	0	0
		10	42000	100%	40	0	0
350 bp	1	0	176	98%	39	1	0
		5	18500	98%	39	0	1
		10	5440	100%	40	0	0
	2	0	1040	91%	19	1	1
		5	17000	100%	40	0	0
		10	6300	100%	40	0	0
1.4 kb	1	0	2420	100%	40	0	0
		5	20000	98%	39	0	1
		10	20000	100%	40	0	0
	2	0	136	100%	40	0	0
		5	8360	100%	40	0	0
		10	8340	100%	40	0	0
	3	0	483	100%	40	0	0
		5	10200	100%	40	0	0
		10	11400	100%	40	0	0
4.3 kb	1	0	714	95%	38	1	1
		5	13000	100%	40	0	0
		10	23300	100%	40	0	0
	2	0	474	100%	40	0	0
		5	4150	98%	39	1	0
		10	10700	95%	38	2	0
	3	0	309	100%	40	0	0
		5	6420	100%	40	0	0
		10	2880	98%	39	1	0

Table S8. Effect of megaprimer concentration on overlap extension cloning efficiency. 20 µL OEC reactions were performed with 25 ng template and 30[a] or 18[b] thermal cycles using the indicated amount of purified megaprimer. [c]Anomalous result.

Mega-primer	Exp. #	fmol	Colonies	Cloning Efficiency	White	Blue	Kan (-)
84 bp	1[a]	100	2480	85%	34	5	1
		250	15100	95%	38	1	1
		500	11900	93%	37	3	0
		750	1600	88%	35	4	1
		1000	9300	88%	35	5	0
	2[a]	100	5690	70%	28	12	0
		250	6380	93%	37	3	0
		500	4710	88%	35	5	0
		750	3470	80%	32	8	0
		1000	1730	80%	32	7	1
350 bp	1[b]	10	299	23%	9	31	0
		33	2420	45%	18	22	0
		100	3330	53%	21	18	1
		330	7640	70%	28	12	0
	2[b]	10	525	15%	6	33	1
		33	2400	38%	15	24	1
		100	7720	45%	18	19	3
		330	4410	43%	17	13	10
		1000	3870	10%	4	2	34
		3330	62	5%	2	15	23
	3[b]	10	89	30%	12	27	1
		33	473	58%	23	17	0
		100[c]	42	10%	4	36	0
		330	9840	63%	25	14	1
		1000	4440	20%	8	15	17
		3330	51	10%	4	7	29
	4[b]	10	331	20%	8	32	0
		33	3360	40%	16	23	1
		100	10900	65%	26	12	2
		330	11600	70%	28	12	0
		1000	1240	38%	15	21	4
		3330	10	10%	1	9	0
1.4 kb	1[a]	10	306	3%	1	37	2
		25	408	18%	7	33	0
		50	861	65%	26	14	0
		100	1450	73%	29	11	0
	2[a]	10	145	20%	8	32	0
		25	19	24%	4	13	0
		50	13	15%	2	11	0
		100	0	n/a	n/a	n/a	n/a

4.3 kb	1[a]	10	251	45%	18	22	0
		25	135	35%	14	26	0
		50	20	21%	5	18	1
		100	0	n/a	n/a	n/a	n/a
	2[a]	10	211	13%	5	37	1
		25	151	5%	2	38	0
		50	57	0	0	39	1
		100	3	0	0	3	0

Table S9. Increased PCR template can generate insert template background in OEC for high megaprimer concentrations. 20 µL OEC reactions were performed with 25 ng template and 18 thermal cycles using the indicated amount of purified 350 bp Gluc megaprimer obtained from a PCR using 5 or 0.5 ng of template.

PCR template	fmol	Colonies	Cloning Efficiency	White	Blue	Kan (-)
5 ng	10	532	40%	16	24	0
	33	1920	38%	15	25	0
	100	3800	58%	23	12	5
	330	2940	50%	20	12	8
	1000	2670	15%	6	6	28
	3330	19	0%	0	6	13
0.5 ng	10	557	18%	7	33	0
	33	2600	43%	17	23	0
	100	5010	60%	24	15	1
	330	2830	48%	19	20	1
	1000	408	40%	16	16	8
	3330	0	n/a	n/a	n/a	n/a

Table S10. Colony number increases with additional cycles of overlap extension. 20 µL OEC reactions were performed with 25 ng template, 100 fmol of purified 350 bp Gluc megaprimer and the indicated number of thermal cycles.

Exp. #	Cycles	Colonies	Cloning Efficiency	White	Blue	Kan (-)
1	14	3380	83%	33	7	0
	16	5170	58%	23	17	0
	18	9550	73%	29	11	0
	20	13100	73%	29	11	0
	22	15100	80%	32	6	2
2	14	4740	40%	16	24	0
	18	8320	53%	21	18	1
	22	14400	60%	24	14	2
	26	28700	65%	26	12	2
	30	33200	78%	31	6	3
3	14	2880	38%	15	23	2
	18	6700	53%	21	18	1
	22	12800	58%	23	16	1
	26	15800	65%	26	14	0
	30	21500	78%	31	9	0

Table S11. Direct comparison of PIPE, SLIC and OEC for increasing insert sizes. Techniques were performed using the optimised conditions, as described in the Materials and Methods.

Insert	Exp. #	Technique	Colonies	Cloning Efficiency	White	Blue	Dead
85 bp	1	PIPE	1680	100%	40	0	0
		SLIC	15100	100%	40	0	0
		OEC	15100	95%	38	1	1
	2	PIPE	806	100%	40	0	0
		SLIC	8740	100%	40	0	0
		OEC	6700	100%	40	0	0
	3	PIPE	2650	98%	39	0	1
		SLIC	77700	100%	40	0	0
		OEC	10600	93%	37	3	0
350 bp	1	PIPE	284	93%	37	2	1
		SLIC	1440	98%	39	1	0
		OEC	15800	85%	34	6	0
	2	PIPE	262	95%	38	1	1
		SLIC	1870	98%	39	0	1
		OEC	11400	78%	31	9	0
	3	PIPE	1030	91%	19	1	1
		SLIC	17000	100%	40	0	0
		OEC	6970	90%	36	0	4
	4	PIPE	176	98%	39	1	0
		SLIC	18500	98%	39	0	1
		OEC	9200	90%	36	1	3
1.4 kb	1	PIPE	705	95%	38	0	2
		SLIC	2760	100%	40	0	0
		OEC	1450	73%	29	11	0
	2	PIPE	183	100%	40	0	0
		SLIC	1580	100%	40	0	0
		OEC	145	20%	8	32	0
4.3 kb	1	PIPE	309	100%	40	0	0
		SLIC	6420	100%	40	0	0
		OEC	251	45%	18	22	0
	2	PIPE	110	95%	38	2	0
		SLIC	1640	100%	40	0	0
		OEC	211	13%	5	37	1

Table S12. Cloning of a 350 bp fragment without quantification or purification. 5 ng of pUC18/Kan pre-cleaved with PstI was amplified by PCR.

[a]5 μL of 350 bp Gluc insert and vector PCR products were combined and digested with DpnI without dilution or purification.

[b]30 μL of insert and vector PCR product were combined, purified and equal volumes treated with T4 for SLIC or left untreated for PIPE.

[c]1 μL of unpurified insert PCR product was used as megaprimer for OEC.

[d]100 fmol of purified megaprimer was used for OEC.

[e]The number of colonies relative to unpurified, unconcentrated PIPE.

Cloning Technique	Cloning Efficiency	Colonies	Fold[e] Increase
PIPE[a] (Unpurified)	95%	226	1
PIPE[b] (Purified)	95%	631	3
SLIC[b] (5 min)	100%	1920	8
SLIC[b] (10 min)	100%	2220	10
OEC[c] (Unpurified)	80%	44	0.2
OEC[d] (Purified)	76%	206	1
