# Supplementary material for: A Practical Comparison of Ligation-Independent Cloning Techniques
Source: PLoS One. 2013 Dec 23;8(12):e83888. doi: 10.1371/journal.pone.0083888 (PMC3871625; doi:10.1371/journal.pone.0083888)
Supplement: File S3 — Supporting Protocols. (PDF) [file pone.0083888.s003.pdf]

PROTOCOLS FOR LIGATION-INDEPENDENT CLONING

Primer design

Normal primer design rules apply. For example, for the insert:

1. Design a 3' end complementary to the insert target 20-25 bp long with a Tm of ~60 °C (E.g. Using modified Breslauer's thermodynamics, http://www.thermoscientificbio.com/webtools/tmc/) ideally ending with a 3' G or C, but avoiding longs runs of G/C. Software such as Primer3, http://primer3.sourceforge.net could also be used.

2. Add ~15 bp of vector sequence to the 5' end for PIPE or SLIC corresponding to the cloning junction. For OEC, a longer ~30 bp tail of target vector sequence is required, ideally with a Tm of 65 °C or more and with a G or C at the 5' end.

The forward primer structure is:
5'-upstream vector sequence-upstream insert sequence-3'

The reverse primer will be the reverse complement (RC) of the vector cloning junction sequence followed by the reverse complement of the insert sequence.

Final reference sequence structure:
5'-downstream insert sequence-downstream vector sequence-3'

Reverse primer structure:
5'-RC downstream vector sequence-RC downstream insert sequence-3'

3. Check the propensity of the primers to form dimers or secondary structures (E.g. Using OligoAnalyzer by Integrated DNA Technologies: http://www.idtdna.com/analyzer/applications/oligoanalyzer/). Structures with a Delta G greater than -6 kcal/mole suggest that redesign may be required, particularly for those at the 3' end. For example, by increasing the length of the 5'-tail.

PIPE cloning without purification

1. Amplify the insert and vector by PCR using a minimum amount of vector plasmid template (0.5 ng template/50 μL reaction) according to manufacturer's instructions with a high fidelity DNA polymerase, such as Phusion Hot Start II High Fidelity DNA Polymerase, but omit the final extension step. An enhancer such as 5% DMSO can be added to increase robustness.

An example thermal cycling program for Phusion:
98 °C 3 min,
(98 °C 30 s 63 °C 30 s 72 °C 45 s/kb) x 35

2. Check the products on an agarose gel for successful amplification. If vector plasmid amplification failed, cut the vector with a restriction enzyme within the multiple-cloning site and repeat the PCR using more template.

If non-specific products are observed, you may wish to optimise the PCR conditions or gel extract the correct fragment for maximum cloning efficiency. For gel extraction of the correct fragment, avoid exposure to damaging short-wave UV light, such as by using methylene blue staining with visible light, or loading duplicate samples, cutting the gel in half between them and marking the transilluminated half as a reference.

3. Combine 5 μL of vector and 5 μL of insert PCR product (or vary accordingly for varying yields by band intensity to get close to a 2:1 molar ratio) with 10 U (0.5 μL) of DpnI and incubate at 37 °C for 1-3 hr. To save time, digestion can be performed during gel electrophoresis.

4. Transform ultracompetent bacteria with the digest mixture up to 10% of the final volume of cells according to manufacturer's instructions. For example, add 2 μL DNA to 18 μL of XL10 Gold cells

PIPE cloning with purification and SLIC using T4 DNA polymerase

1. Perform PCR as above.

2. Analyse products by agarose gel electrophoresis as above.

3. Purify products using a single PCR purification column with a minimal elution (~30 μL) volume.

Alternatively, purify separately, quantify by spectroscopy and combine 0.025 pmol vector (~50-100 ng) and 0.0625 pmol insert (~50-200 ng) per 10 μL.

4. Make up with 1x CutSmart Buffer or equivalent and digest with 10 U (0.5 μL) of DpnI/10 μL reaction volume for 1-3 hr at 37 °C.

5. Separate into three fractions and incubate 10 μL samples for 0 (PIPE), 5 or 10 min at 25 °C with 0.75 U (0.25 μL) T4 DNA polymerase, then place on ice for 10 min.

6. Transform bacteria as above.

Overlap extension cloning

1. Perform PCR as above.

2. Analyse products by agarose gel electrophoresis as above.

If non-specific products or primer-dimers are present it is essential to remove them by PCR optimisation, gel extraction as above, or T4 treatment.

3. Column purify megaprimer and quantify by spectroscopy.

For products <500 bp, 1 μL of PCR product can be used directly for thermal cycling without purification. Reduced efficiencies may be observed for larger fragments.

4. Perform overlap extension linear amplification.

The optimal megaprimer concentration to use is: <350 bp, ~100-300 fmol (5-15 nM); <1.5 kb ~50-100 fmol (2.5-5 nM); >1.5 kb, ~25-50 fmol (1.25-2.5 nM).

To remove primer-dimers with T4 DNA polymerase, treat a 10 μL fraction of 1x SmartCut buffer containing ten-fold the optimum megaprimer in 10 μL, incubate with 3 U (1 μL) of T4, incubate for 30 min at 25 °C and immediately use 5 μL for overlap extension.

Perform thermal cycling according to manufacturer's instructions with a high fidelity, non-strand-displacing DNA polymerase, such as Phusion Hot Start II High Fidelity DNA Polymerase, a 20 μL reaction volume, 25 ng of plasmid template and 5% DMSO.

An example thermal cycling program for Phusion:
72 °C 5 min (to blunt the megaprimer),
98 °C 3 min,
(98 °C 30 s 63 °C 30 s 72 °C 45 s/kb) x 30

5. Dilute 5 uL of product with 5 uL CutSmart Buffer (to maximise DpnI activity and reduce inhibition by PCR buffer) and 20 U (1 μL) DpnI and incubated for 1-3 hr at 37 °C.

6. Transform bacteria as above.
